# The effects of long‐term continuous positive airway pressure on apnea–hypopnea index change following short‐term that withdrawal in patients with obstructive sleep apnea

**DOI:** 10.1111/crj.13488

**Published:** 2022-04-24

**Authors:** Longlong Wang, Minxia Pan, Qiong Ou

**Affiliations:** ^1^ The Second School of Clinical Medicine Southern Medical University Guangzhou China; ^2^ Sleep Center, Department of Pulmonary and Critical Care Medicine Guangdong Provincial People's Hospital, Guangdong Academy of Medical Sciences; Guangdong Provincial Geriatrics Institute Guangzhou China

**Keywords:** cohort study, continuous positive airway pressure, early diagnosis, severity of illness index, sleep apnea, obstructive

## Abstract

The effect of long‐term continuous positive airway pressure (CPAP) treatment on apnea–hypopnea index (AHI) after CPAP withdrawal remains unclear, especially in obstructive sleep apnea (OSA) patients screened from the population. To examine that, 1241 civil servants who participated in the annual physical examination were screened for OSA between September and December 2017. Screened OSA firstly underwent 1‐week CPAP adherence assessment. Then, patients with good CPAP adherence would be freely provided CPAP to continued treatment. All OSA patients were followed for 2 years. At study end, all OSA patients underwent home sleep testing (HST) again within 1 week of CPAP withdrawal. The effect of 2‐year CPAP treatment on OSA severity was investigated by using linear regression and multinominal logistic regression. In total, 103 OSA patients were screened, including 41 cases (39.8%) in CPAP treatment group and 62 cases (60.2%) in non‐CPAP treatment group. At 2‐year follow‐up, compared with baseline, in CPAP treatment group, following CPAP withdrawal, a significant decrease in AHI was observed in patients with severe OSA (*P* = 0.014); in non‐CPAP treatment group, a significant increase in AHI was observed in patients with moderate OSA (*P* = 0.028). After adjustment for confounding factors, multivariate linear regression showed that △AHI was negatively associated with CPAP treatment (*β* = −4.930, 95% confidence interval [CI] [−9.361, −0.500], *P* = 0.030). Multinominal logistic regression showed that the AHI of patients not treated with CPAP tended to be unchanged or worsened with the AHI improvement group as a reference (OR [odds ration] [95% CI], 4.555 [1.307, 15.875], *P* = 0.017; 6.536 [1.171, 36.478], *P* = 0.032). In conclusion, active OSA screening and long‐term CPAP intervention may improve the severity of severe OSA patients following short‐term CPAP withdrawal in the general population.

## Graphical abstract

In general population, active OSA screening and CPAP intervention, a new model for the management of OSA, are feasible. Population‐based OSA screening and long‐term CPAP intervention may improve the severity of severe OSA patients following short‐term CPAP withdrawal.

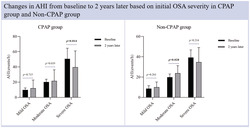

## 1 INTRODUCTION

Obstructive sleep apnea (OSA), characterized by repetitive episodes of complete or partial upper airway obstructive, leads to intermittent hypoxia and sleep architecture disturbance. OSA is a risk factor of multisystem disease, such as hypertension, diabetes mellitus, coronary heart disease, and stroke.^1^, ^2^ In recent years, the prevalence of OSA has been increasing in parallel with the obesity epidemic.^3^ Previous studies have shown that untreated OSA has an increased risk of cardiovascular morbidity and all‐cause mortality and has become a public health concern.^4^, ^5^

Continuous positive airway pressure (CPAP) is currently the first‐line therapeutic strategy for OSA.

Significant effectiveness with CPAP therapy was observed in improving excessive daytime sleepiness (EDS), sleep‐related quality of life, and blood pressure.^6^ However, different studies have reported inconsistent results regarding the effect of CPAP on the cardiovascular outcomes in OSA patients.^7^, ^8^, ^9^, ^10^ Variability with OSA severity at follow‐up may be one of the key factors behind these conflicting results. Currently, OSA severity is determined by apnea–hypopnea index (AHI). The effect of CPAP treatment on AHI after CPAP withdrawal is unclear, especially in OSA patients screened from the population. Many previous studies have investigated the natural evolution of OSA severity,^11^, ^12^, ^13^ but few studies focus on the effect of CPAP treatment on that in the general population. Active OSA screening and early intervention in the general populations may be an innovative model to decrease OSA severity and improve cardiovascular outcomes.

In addition, many OSA patients do not tolerate CPAP therapy. In clinical practice, what puzzles clinicians and patients is that whether once CPAP is prescribed, the patients need to use it forever, that is, a key factor impacting the acceptance in CPAP therapy for OSA patients. Current studies have focused only on improving adherence to CPAP therapy, but few studies answer this common clinical question.

Therefore, based on active OSA screening and intervention in the general populations, the aim of the study was to examine the impact of long‐term CPAP therapy on AHI after CPAP withdrawal.

## 2 MATERIALS AND METHODS

### 2.1 Study population

The subjects were a group of civil servants populations who participated in the annual health examination from September to December 2017 in Guangzhou, Guangdong Province, China. The cohort study consisted of demographic, anthropometric, medical history, sleep‐related questionnaires, OSA diagnosis, CPAP treatment, regular follow‐up, and other related information.

### 2.2 OSA screening and diagnosis

First, Berlin questionnaire^14^ was used to identify high‐risk OSA patients in the civil servants populations. Then, a home sleep testing (HST, ApneaLink Air, ResMed, Australia) was performed for high‐risk OSA patients. The monitoring parameters included nasal airflow, nocturnal oxygen saturation, heart rate, and respiratory effort. Simultaneous recording of nasal airflow and nocturnal oxygen saturation should take more than 4 h.

An obstructive apnea was defined as a decrease in respiratory airflow by ≥90% of pre‐event baseline for ≥10 s with continued respiratory effort; an obstructive hypopnea is defined as a decrease in respiratory airflow by ≥30% of pre‐event baseline for ≥10 s followed by a decrease in SaO_2_ of ≥3%. The AHI was defined as the number of apneas and hypopneas per hour of sleep. Patients were diagnosed as OSA based on an AHI of ≥5 events/h.

Based on AHI, OSA was classified as mild (5 ≤ AHI < 15), moderate (15 ≤ AHI < 30), or severe (AHI ≥ 30). Improvement or worsening of initial AHI was defined as an increase or decrease of ≥25% of AHI.^15^ The Epworth Sleepiness Scale (ESS) of ≥9 was considered as EDS.

### 2.3 CPAP Treatment

Screened OSA patients firstly underwent 1‐week CPAP adherence assessment based on their willingness. OSA patients with good CPAP adherence^16^ (defined as ≥4 h/night of CPAP use) would be freely provided auto‐CPAP to continue treatment. Patients with poor compliance were required to return the CPAP device. All subjects received standardized sleep hygiene and healthy lifestyle education.

### 2.4 Follow‐up

All OSA patients were followed up regularly for 2 years at 1, 3, 6, 18, 12, 18, and 24 months. At study end, all OSA patients underwent HST test again within 1 week of CPAP withdrawal. The primary endpoint was AHI, and the secondary endpoints included new‐onset diseases, body mass index (BMI), and oxygen desaturation index (ODI).

### 2.5 Statistical analysis

As the Shapiro–Wilk test showed some parameters were not normally distributed, the nonparametric Wilcoxon signed rank test was used to compare the mean baseline AHI, BMI, ODI, mean SaO_2_, and Tsat90 with that of end of follow‐up. Categorical variables were analyzed by using a chi‐square test. Multivariate linear regression was performed to determine the effect of CPAP treatment on △AHI (△AHI = AHI_2 years later_ − AHI_baseline_) after adjustment for other variables. Multinominal logistic regression was conducted in order to further identify the effect of CPAP therapy on OSA severity according to three levels of AHI change (improved, unchanged, and worsened). All analyses were performed with SPSS 25.0 software (SPSS, Chicago, IL). A *P* value of <0.05 was considered to be statistically significant.

## 3 RESULTS

**FIGURE 1:**
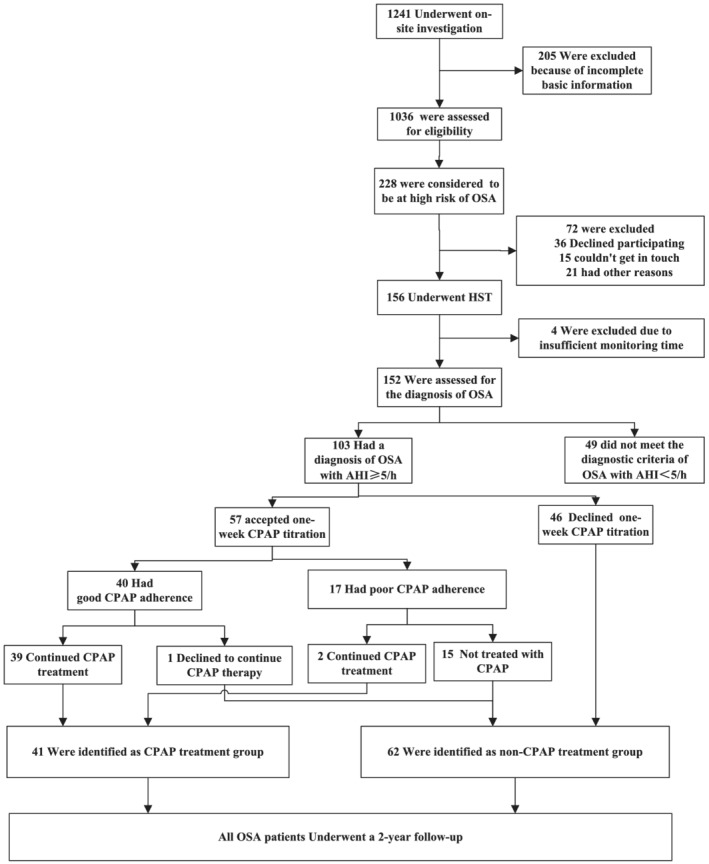
Flowchart of the study. AHI, apnea–hypopnea index; CPAP, continuous positive airway pressure; OSA, obstructive sleep apnea

In total, 1241 civil servants in service underwent on‐site investigation, and 205 were excluded because of incomplete basic information. The Berlin questionnaire identified 228 patients at high risk for OSA among 1036 civil servants, of which 156 accepted HST monitoring, and four were excluded due to insufficient monitoring time. Finally, 103 were diagnosed with OSA, among them, 57 accepted 1‐week CPAP adherence assessment. Forty patients had good 1‐week CPAP compliance, of whom 39 continued CPAP treatment; 17 with poor adherence, and two continued CPAP treatment. Therefore, 41 were identified as CPAP treatment group, and 62 were identified as non‐CPAP treatment group. The flowchart of the study is shown in Figure 1.

The average age of the OSA patients was 44.8 ± 8.3 years, and 96.1% were male. Characteristics of the total group and for CPAP and non‐CPAP group at baseline and follow‐up are reported in Table 1. In the CPAP group, a significant decrease in AHI was found (*p* = 0.04), and a significant improvement in mean SaO_2_, Tsat90, and ODI was also reported. In non‐CPAP group, we reported an insignificant increase in AHI (*p* = 0.120). During the 2 years of follow‐up, we reported one new‐onset diabetes mellitus for CPAP group, three new‐onset hypertension, and one diabetes mellitus for non‐CPAP group.

**TABLE 1:** Clinical, anthropometric, and sleep parameters for total group, CPAP treatment, and non‐CPAP treatment group at baseline and at follow‐up

	OSA (*n* = 103)			CPAP group (*n* = 41)			Non‐CPAP group (*n* = 62)		
	Baseline	2 years later	*P*	Baseline	2 years later	*P*	Baseline	2 years later	*P*
Age (years)	44.8 ± 8.3			45.7±7.8			44.1±8.6		
Gender (M/F)	99/4			41/0			58/4		
AHI (events/h)	22.9 ± 17.2	22.0 ± 16.8	0.574	33.0 ± 19.4	28.9 ± 20.3	**0.04**	16.2 ± 11.4	17.4 ± 12.1	0.120
BMI (kg/m^2^)	26.3 ± 2.5	26.2 ± 2.6	0.433	26.7 ± 2.8	26.8 ± 3.3	0.910	26.0 ± 2.2	25.9 ± 2.1	0.344
Mean SaO_2_ (%)	93.3 ± 1.8	93.9 ± 1.6	**0.027**	92.8 ± 2.4	93.8 ± 1.8	**0.021**	93.7 ± 1.2	93.9 ± 1.5	0.416
ODI (events/h)	20.9 ± 16.1	20.2 ± 16.1	0.421	30.1 ± 18.5	26.4 ± 20.0	**0.046**	14.9 ± 10.7	16.2 ± 11.4	0.340
Tsat90 (%)	10.1 ± 12.6	8.8 ± 11.3	0.205	15.5 ± 15.7	11.6 ± 13.6	**0.038**	6.6 ± 8.7	7.0 ± 9.1	0.619
Hypertension, *n* (%)	50 (48.5)	53 (51.5)	0.676	25 (61)	25 (61)	1.000	25 (40.3)	28 (45.2)	0.586
Diabetes mellitus, *n* (%)	4 (3.9)	6 (5.8)	0.517	2 (4.9)	3 (7.3)	1.000	2 (3.2)	3 (4.8)	1.000
Coronary heart disease, *n* (%)	4 (3.9)	4 (3.9)	1.000	1 (2.4)	1 (2.4)	1.000	3 (4.8)	3 (4.8)	1.000

Abbreviations: AHI, apnea–hypopnea index; BMI, body mass index; CPAP, continuous positive airway pressure; ODI, oxygen desaturation index; OSA, obstructive sleep apnea; SaO_2_, oxygen saturation; Tsat90, percentage of night time spent with an oxygen saturation below 90%.

Of the 103 patients with OSA, 41 were diagnosed as mild (39.8%), 34 moderate (33.0%), and 28 severe (27.2%). Table 2 shows the changes in AHI from baseline to 2 years later based on initial OSA severity.

**TABLE 2:** Changes in AHI from baseline to 2 years later based on initial OSA severity

	OSA (*n* = 103)			CPAP group (*n* = 41)			Non‐CPAP group (*n* = 62)		
	Baseline	2 years later	*P*	Baseline	2 years later	*P*	Baseline	2 years later	*P*
Mild	8.8 ± 2.8	10.2 ± 6.0	0.416	9.8 ± 2.6	12.0 ± 10.8	0.715	8.7 ± 2.8	10.0 ± 5.2	0.261
Moderate	20.0 ± 3.4	22.8 ± 11.4	0.270	20.2 ± 3.7	21.7 ± 14.5	0.619	19.8 ± 3.2	23.9 ± 7.3	**0.028**
Severe	47.0 ± 13.3	38.2 ± 19.2	**0.008**	50.6 ± 14.0	39.7 ± 21.3	**0.014**	39.3 ± 7.5	34.8 ± 14.3	0.214

Abbreviations: AHI, apnea–hypopnea index; CPAP, continuous positive airway pressure; OSA, Obstructive sleep apnea.

Compared with baseline, in CPAP group, following CPAP withdrawal, a significant decrease in AHI was observed for patients with severe OSA, while no significant change in AHI for patients with mild and moderate OSA; in non‐CPAP group, the AHI increased significantly for patients with moderate OSA, while no significant change for patients with mild and severe OSA.

Univariate linear regression showed that the △AHI was significantly correlated with △BMI, CPAP treatment, ESS, lowest SaO_2_, and time SaO_2_ < 90%. Variables with a *P* value of <0.05, plus age, were retained for inclusion in the multivariable linear regression models (lowest SaO_2_ and time SaO_2_ < 90% were excluded due to significantly correlated with CPAP therapy). The results showed that △AHI was significantly negatively correlated with CPAP treatment after adjusting for age, △BMI, ESS (*β* = −4.930; 95% confidence interval [CI] [−9.361, −0.500]; *P* = 0.030). Results of the linear regression are shown in Table 3.

**TABLE 3:** Predictors of AHI change(△AHI) by linear regression analysis

	Univariate linear regression		Multiple variable linear regression	
Predictors	*β* (95% CI)	*P*	*β* (95% CI)	*P*
Age (years)	0.155 (−0.128, 0.439)	0.280	−0.183 (−0.447, 0.081)	0.173
△BMI	1.796 (0.307, 3.284)	**0.019**	2.031 (0.626, 3.436)	**0.005**
CPAP treatment	5.369 (0.690, 10.047)	**0.025**	−4.930 (−9.361, −0.500)	**0.030**
ESS	0.686 (0.173, 1.199)	**0.009**	−0.723 (−1.214, −0.232)	**0.004**
Hypertension	3.728 (−0.912, 8.368)	0.114		
Neck circumference (cm)	0.338 (−0.643, 1.319)	0.496		
Lowest SaO_2_ (%)	0.518 (0.216, 0.820)	**0.001**		
Time SaO_2_ < 90% (min)	−0.097 (−0.145, −0.049)	**<0.001**		

*Note*: △AHI = AHI_2 years later_ − AHI_baseline_; △BMI = BMI_2 years later_ − BMI_baseline_.

Abbreviations: AHI, apnea–hypopnea index; BMI, body mass index; CI, confidence interval; CPAP, continuous positive airway pressure; ESS, Epworth Sleepiness Scale; SaO_2_, oxygen saturation.

A cut‐off value of 25% was used to define improvement or worsening of initial AHI. In total, 24 (23.3%) OSA patients improved their initial AHI, 61 (59.2%) remained unchanged, and 18 (17.5%) worsened.

Univariate multinomial logistic regression showed that the change in AHI was significantly associated with initial OSA severity, CPAP treatment, and mean SaO_2_ at baseline. Based on a threshold *P* value < 0.05, we conducted multivariate multinomial logistic regression including these variables and age.

Adjusting for these potential confounding factors, the results showed that the AHI of OSA patients who were not treated with CPAP tended to be unchanged or worsened with the AHI improvement group as a reference (OR [odds ratio] [95% CI], 4.555 [1.307, 15.875], *P* = 0.017; 6.536 [1.171, 36.478], *P* = 0.032). Tables 4 and 5 show the results of multinomial logistic regression analysis.

**TABLE 4:** Predictors of OSA severity change by univariate multinomial logistic regression

Predictors	Stable		Worsened	
	OR (95% CI)	*P*	OR (95% CI)	*P*
Age (years)	1.042 (0.981, 1.107)	0.179	0.934 (0.863, 1.010)	0.086
Baseline BMI	1.002 (0.830, 1.210)	0.983	0.891 (0.683, 1.162)	0.395
△BMI	1.138 (0.813, 1.593)	0.450	1.328 (0.883, 1.998)	0.173
OSA severity				
Mild	1.667 (0.555, 5.010)	0.363	4.444 (0.740, 26.678)	0.103
Moderate	2.625 (0.748, 9.210)	0.132	8.000 (1.215, 52.693)	**0.031**
Severe	Reference		Reference	
CPAP treatment				
No	3.175 (1.191, 8.465)	**0.021**	4.333 (1.156, 16.248)	**0.030**
Yes	Reference		Reference	
EDS				
No	1.735 (0.661, 4.551)	0.263	2.962 (0.752, 11.666)	0.121
Yes	Reference		Reference	
Mean SaO_2_(%)	1.078 (0.843, 1.379)	0.548	1.676 (1.079, 2.602)	**0.021**
Lowest SaO_2_(%)	1.041 (0.979, 1.108)	0.200	1.088 (0.992, 1.192)	0.073
Time SaO_2_< 90% (min)	0.998 (0.988, 1.007)	0.636	0.978 (0.956, 1.000)	0.050

*Note*: △BMI = BMI_2 years later_ − BMI_baseline_.

Abbreviations: BMI, body mass index; CI, confidence interval; CPAP, continuous positive airway pressure; EDS, excessive daytime sleepiness; OR, odds ration; OSA, obstructive sleep apnea; SaO_2_, oxygen saturation.

**TABLE 5:** Predictors of OSA severity change by multivariate multinomial logistic regression

Predictors	Stable		Worsened	
	OR (95% CI)	*P*	OR (95% CI)	*P*
Age (years)	1.055 (0.988, 1.127)	0.112	0.953 (0.874, 1.038)	0.267
OSA severity				
Mild	0.995 (0.219, 4.527)	0.995	0.715 (0.071, 7.216)	0.776
Moderate	2.930 (0.621, 13.833)	0.175	3.765 (0.395, 35.899)	0.249
Severe	Reference		Reference	
CPAP treatment				
No	4.555 (1.307, 15.875)	**0.017**	6.536 (1.171, 36.478)	**0.032**
Yes	Reference		Reference	
Mean SaO_2_ (%)	0.943 (0.687, 1.295)	0.716	1.369 (0.790, 2.372)	0.263

Abbreviations: CI, confidence interval; CPAP, continuous positive airway pressure; OR, odds ration; OSA, obstructive sleep apnea; SaO_2_, oxygen saturation.

## 4 DISCUSSION

The primary finding of this study is that population‐based OSA screening and CPAP intervention can significantly delay the progression of OSA severity, especially in patients with severe OSA. In addition, a significant improvement was also found in the nocturnal hypoxia in OSA patients treated with CPAP.

There exists an increased prevalence of OSA in recent years in tandem with the rise in obesity globally.

However, available data indicate that a large number of suspected OSA patients are undiagnosed and untreated according to the current mode of voluntarily seeking medical care.^17^ Untreated OSA is related to an increased risk of a wide variety of comorbidities and healthcare costs. New Healthy People 2020 guidelines were released by the Office of Disease Prevention and Health Promotion, and “sleep health” was regarded as a national priority, whereby one of primary objectives is increasing the proportion of suspected OSA who seek medical attention.^18^ Nevertheless, due to an insufficient recognition of the disorder, the proportion actively seeking medical care remains low for high‐risk OSA patients.

There is no definitive recommendation regarding whether OSA screening and intervention should be performed in the general population. US Preventive Services Task Force (USPSTF) has considered that insufficient evidence was existing on the benefits and harms of screening for OSA in asymptomatic adults.^19^ However, the potential benefits of screening for OSA in the general populations seem to be obvious. CPAP treatment for screened OSA patients can not only improve EDS and quality of life^6^ but also delay the progression of OSA severity. Based on the high prevalence of OSA, low diagnosis and treatment rate, high disease burden,^20^, ^21^ and the effectiveness of CPAP treatment, the mode of screening for OSA in general populations, and performing intervention are likely to be worth generalizable.

Previous longitudinal studies have demonstrated that weight change plays a key role in triggering the progression of OSA severity. A study from the Wisconsin Sleep Cohort showed a 10% of weight gain led to a 32% of increase in AHI, while a 10% of weight loss led to a 26% of decrease in AHI^22^; on the other hand, no AHI changes were observed in OSA patients who remained stable weight.^23^ The present study showed that CPAP therapy independently predicts the change of OSA severity after adjusting for confounding of weight change. Nevertheless, the underlying mechanism for this is not strictly clear. To determine whether CPAP treatment is able to change upper airway morphology in OSA patients, Ryan and colleagues^24^ conducted upper airway MRI scans on five moderate‐to‐severe OSA patients after 4–6 weeks of CPAP treatment. The results showed a reduction in pharyngeal oedema and a decrease in tongue size, resulting in the increase of pharyngeal volume. This may in part explain the result that CPAP therapy reduced the AHI, especially for patients with severe OSA.

Rossi and colleagues^25^ found a third of CPAP‐treated patients with moderate‐to‐severe OSA did not experience significant recurrence of OSA after CPAP withdrawal for four nights, and almost half of them did not experience a return of OSA even after 2‐week off CPAP. In the present study, following CPAP withdrawal, seven patients with prior moderate‐to‐severe OSA treated with CPAP changed into mild OSA, and one mild OSA with CPAP therapy had a normal AHI at 2‐year follow‐up. Hence, we believe that in some patients with OSA treated with CPAP, short‐term CPAP withdrawal may be feasible and may even be converted to a simpler approach, such as oral appliance.

Our study shows that long‐term CPAP therapy can delay the progression of OSA severity, especially for severe OSA with a significant reduction in AHI after CPAP withdraws. Previous study has reported that in a subset of OSA patients treated with CPAP, OSA is temporarily “cured” for up to 2 weeks.^25^ These results suggest that some recognition perhaps should be changed that once CPAP is prescribed, the patients need to use it forever. This has practical applications in enhancing the beliefs of treating disease and improving adherence to CPAP therapy for OSA patients, especially in the mode of active screening for OSA and intervention.

CPAP therapy significantly decreased AHI in patients with severe OSA; however, as previous studies, a decrease in AHI was also found in untreated severe OSA patients,^11^, ^12^ which may be related to healthy lifestyle education. The lack of AHI increases in severe patients suggesting a ceiling effect may exist; namely, if AHI reaches a critical point, the mechanism of body protection may be activated, thus sparing them from the effect of severe nocturnal hypoxemia.

This study was a population‐based cohort study. The potential strength is that portable home sleep monitoring was performed on all OSA patients at the end of follow‐up, thus reducing the chance of selection bias. However, our study also has some limitations. First, due to the variability of AHI, which is affected by sleep position and the percentage of sleep time in rapid eye movement (REM), the severity of OSA may be misclassified, especially for patients with mild and moderate OSA.^26^, ^27^, ^28^ Second, the majority of OSA patients in this study were male (96.1%); the influence of gender on the study outcomes has not been evaluated, suggesting that more female patients with OSA should be included in future studies. Third, CPAP treatment was not randomized to patients with OSA. In addition, we did not evaluate the effect of other therapies, such as oral appliances, on disease severity in OSA patients who did not receive CPAP therapy. Future studies should explore the impact of multiple interventions on OSA severity in patients who have been screened for OSA from the population.

In conclusion, the present study provides a new model for the management of OSA, namely, active OSA screening and intervention in general population. Our results have obvious clinical implications in enhancing the beliefs of treating disease and improving adherence to CPAP therapy for OSA patients, especially in the mode of active screening for OSA and intervention.

## CONFLICT OF INTEREST

The authors have no conflicts of interest to disclose.

## AUTHOR CONTRIBUTIONS

Qiong Ou designed the study; Minxia Pan performed the study and data collection; Longlong Wang steered literature search, statistical analysis, and drafted the manuscript; Qiong Ou reviewed and approved the submission of the manuscript.

## ETHICS STATEMENT

This study was approved by the Ethics Committee of Guangdong Provincial People's Hospital (2017244H). All subjects signed an informed consent form and agreed to participate in the study.

## Data Availability

The data that support the findings of this study are available on request from the corresponding author.
